# Medullary Sponge Kidney and Testicular Dysgenesis Syndrome: A Rare Association

**DOI:** 10.1155/2014/841781

**Published:** 2014-02-18

**Authors:** Stefano Masciovecchio, Pietro Saldutto, Giuseppe Paradiso Galatioto, Carlo Vicentini

**Affiliations:** Department of Life, Health & Environmental Sciences, University of L'Aquila, “Giuseppe Mazzini” General Hospital, Teramo, Italy

## Abstract

The medullary sponge kidney is also known as Lenarduzzi's kidney or Cacchi and Ricci's disease from the first Italian authors who described its main features. A review of the scientific literature underlines particular rarity of the association of MSK with developmental abnormalities of the lower urinary tract and genital tract such as hypospadias and bilateral cryptorchidism. The work presented is the only one in the scientific literature that shows the association between the medullary sponge kidney and the testicular dysgenesis syndrome. A question still remains unanswered: are the MSK and TDS completely independent malformation syndromes occurring, in this case, simultaneously for a rare event or are they different phenotypic expressions of a common malformative mechanism? In the future we hope that these questions will be clarified.

## 1. Introduction

The medullary sponge kidney (MSK), also known as Lenarduzzi's kidney or Cacchi and Ricci's disease from the first Italian authors who described its main features [1], is an uncommon renal malformation that usually occurs with nephrocalcinosis and urolithiasis. Less frequently urinary acidification, concentration defects of the urine, and precalyceal duct ectasias can be observed. MSK is generally considered to be a congenital disorder even if there is no clear scientific evidence demonstrating the unique congenital nature of the disease. The condition can be associated with other renal and/or extrarenal developmental abnormalities that characterize, in some cases, specific complex genetic syndromes [2]. A review of the scientific literature underlines particular rarity of the association of MSK with developmental abnormalities of the lower urinary tract and genital tract such as hypospadias and bilateral cryptorchidism. We report the case of MSK associated with testicular dysgenesis syndrome (TDS).

## 2. Case Report

**Figure 1:**
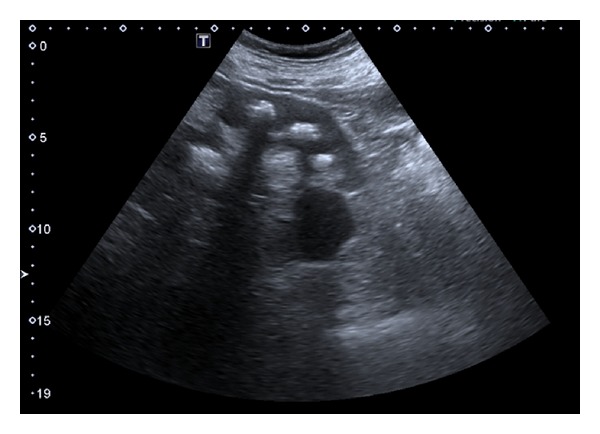
Multiple hyperechoic elements with shadow back.

**Figure 2:**
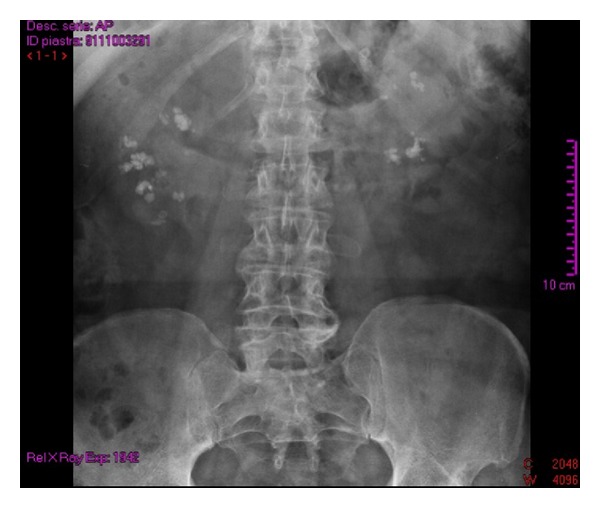
Appearance on “bouquet of flowers.”

**Figure 3:**
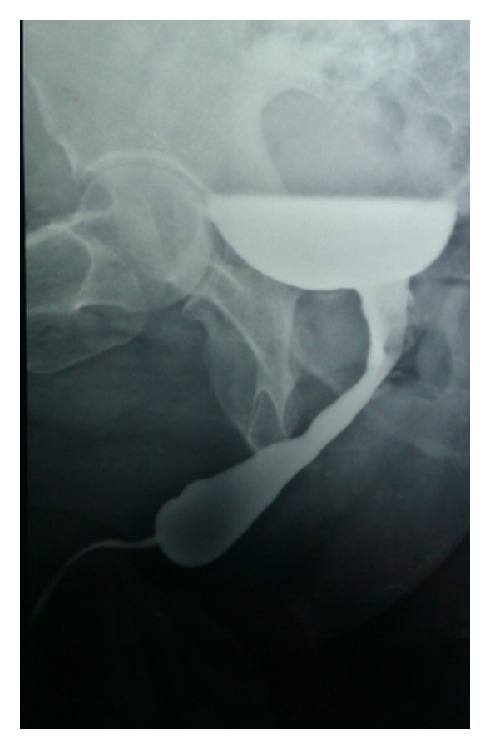
Stricture of the distal anterior urethra.

We observe a 67-year-old man for severe LUTS (IPSS questionnaire score of 23). The patient's medical history reveals right orchidopexy (in childhood) for cryptorchidism with simultaneous contralateral simple orchiectomy performed for unknown reasons, a history of recurrent renal colic (from at least 50 years) always treated with automedication, and infertility. Physical uroandrologic examination shows the presence of glandular hypospadias with slightly painful anterior urethra, normal right didymus and epididymis, and, in accordance with medical history, uninhabited left hemiscrotum. The digital rectal exam shows a prostate volume slightly increased with characters related to adenomatous growth. The blood count, laboratory indices of renal functionality, the hydroelectrolyte balance, and urine examination appear normal. The uroflowmetry tracing appears classically restrictive and the postvoid residual not significant. Renal ultrasonography performed during the office visit shows the presence of bilateral multiple hyperechoic elements with posterior acoustic shadow amenable to urolithiasis and no hydroureteronephrosis (Figure 1). A pathognomonic appearance on “bouquet of flowers” is evident in a kidney-bladder radiograph that the patient brings into view (Figure 2). Subsequently, the patient has been subjected to spiral uro-CT to obtain a morphofunctional evaluation of kidney and, also, to get a visualization of the upper urinary tract that has, however, showed no abnormality. The study of the lower urinary tract visualized by retrograde and voiding urethrocystography shows a short but tight stricture of the distal anterior urethra and a proximal ectasia to the stenosis (Figure 3). The scrotal ultrasound shows normal echogenicity of didymus. The series of blood tests and urine laboratory tests performed in order to assess the phosphocalcium homeostasis are normal. We subjected the patient to internal urethrotomy according to the technique of Sachse with complete resolution of symptomatological condition.

## 3. Discussion

The MSK is a malformative disease that can be classified as a developmental disorder and its pathogenesis should be sought in one of the numerous, complex developmental steps characterizing renal morphogenesis. Differentiation signals originating at the “ureteric bud-metanephric blastema” interface are necessary to coordinate the complex phenomena occurring in nephrogenesis, and among them a pivotal role is played by the glial cell line-derived neurotrophic factor [3] and its receptor RET (receptor tyrosine kinase) [4]. MSK can occur in association with renal developmental anomalies and tumours, such as Wilms' tumour, horseshoe kidney, and contralateral congenital small kidney, and occasionally with pyeloureteral abnormalities or hypertrophic disorders like the Beckwith-Wiedemann syndrome and congenital hemihypertrophy. It may also be associated with liver disorders, such as congenital dilation of the intrahepatic bile ducts (Caroli's disease) and hepatic fibrosis [2, 5, 6]. Gunay-Aygun et al. recently suggested that MSK could be a ciliopathy, based on the observation that MSK has been reported in association with conditions determined by nonmotile cilia disorders such as Young's syndrome [7]. Cryptorchidism and hypospadias are congenital malformations with prevalence in the general population, respectively, equal to 0.05–0.4% and to 0.03–13.4%. Their incidence is increasing in recent decades [8]. These malformations are characterized by common risk factors such as prematurity, low gestational weight, and low birth weight. Also maternal exposure to cigarette smoking, diabetes mellitus, and gestational diabetes are known as risk factors [9]. Recently it has been shown that maternal exposure to exogenous environmental factors (herbicides, fungicides, insecticides, phthalates, etc.) is able to interfere with the synthesis, transport, function, and elimination of sex hormones which may determine an alteration of fetal gonadal cells and, therefore, urogenital defects [10, 11]. This hypothesis could explain the onset of TDS, characterized by simultaneous presence of cryptorchidism and hypospadias as well as seminal disorders and testicular tumors [12, 13]. The work presented is the only one in the scientific literature that shows the association between the MSK and TDS. We believe that the clinical history of the patient and the imaging are sufficient for making a diagnosis of MSK even in the absence of blood and urinary laboratory abnormalities [14]. In our opinion, the presence of LUTS and the restrictive uroflowmetry tracing are caused by stricture of the urethra which is evident at the RX urethrocystography. We believe that the stricture is secondary to urethral injury due to spontaneous expulsion of small stone and not to congenital condition. We also believe that the patient is suffering from TDS with simultaneous presence of cryptorchidism and hypospadias. The testicular palpation and scrotal ultrasound ruled out the presence of a concomitant testicular cancer. A question still remains unanswered: are the MSK and TDS completely independent malformation syndromes occurring, in this case, simultaneously for a rare event or are they different phenotypic expressions of a common malformative mechanism? In the future we hope that these questions will be clarified.

## 4. Conclusion

The MSK is a congenital renal anomaly that may be related to extrarenal malformations and sometimes even to complex genetic syndromes [2]. We describe, for the first time in the literature, a case of MSK associated with TDS. It remains an open question whether these conditions are related to each other or are separate events occurring simultaneously in the same patient.
